# Does the Nucleoid Determine Cell Dimensions in *Escherichia coli*?

**DOI:** 10.3389/fmicb.2019.01717

**Published:** 2019-08-06

**Authors:** Arieh Zaritsky, Waldemar Vollmer, Jaan Männik, Chenli Liu

**Affiliations:** ^1^Faculty of Natural Sciences, Ben-Gurion University of the Negev, Beersheba, Israel; ^2^Centre for Bacterial Cell Biology, Institute for Cell and Molecular Biosciences, Newcastle University, Newcastle upon Tyne, United Kingdom; ^3^Department of Physics & Astronomy, The University of Tennessee, Knoxville, Knoxville, TN, United States; ^4^Shenzhen Institute of Synthetic Biology, Shenzhen Institutes of Advanced Technology, Chinese Academy of Sciences, Shenzhen, China

**Keywords:** cell cycle and dimensions, nucleoid structure and complexity, nutritional shifts, transertion, eclipse, physical effector

## Abstract

Bacillary, Gram-negative bacteria grow by elongation with no discernible change in width, but during faster growth in richer media the cells are also wider. The mechanism regulating the change in cell width *W* during transitions from slow to fast growth is a fundamental, unanswered question in molecular biology. The value of *W* that changes **in** the divisome and **during** the division process only, is related to the nucleoid complexity, determined by the rates of growth and of chromosome replication; the former is manipulated by nutritional conditions and the latter—by thymine limitation of *thyA* mutants. Such spatio-temporal regulation is supported by existence of a minimal possible distance between successive replisomes, so-called eclipse that limits the number of replisomes to a maximum. Breaching this limit by slowing replication in fast growing cells results in maximal nucleoid complexity that is associated with maximum cell width, supporting the notion of Nucleoid-to-Divisome signal transmission. Physical signal(s) may be delivered from the nucleoid to assemble the divisome and to fix the value of *W* in the nascent cell pole.

## Introduction

Rod-shaped bacteria such as *Escherichia coli* grow by elongation without a discernible change in width (Trueba and Woldringh, 1980; Taheri-Araghi et al., 2014), but when growing faster in richer media (at a constant temperature) the cells are both, longer and wider (Schaechter et al., 1958; Zaritsky, 1975a). The nature of the mechanism that regulates the change in cell width during a transition from slow to fast growth, so-called nutritional shift-up, is a fundamental but still unanswered question in bacteriology (Zaritsky and Woldringh, 2015). In this brief *Perspective*, putative mechanisms to control cell width during shift-up and other transitions are discussed, with perspective for future research to test the predictions emanating from new, bold ideas.

Most mutants affecting cell dimensions involve the biosynthetic pathway of peptidoglycan (PG)—the shape-maintaining macromolecule and the bacterial cytoskeleton, including the actin-like MreB and the tubulin-like FtsZ, and their associated cell morphogenesis proteins (Typas et al., 2012). MreB is essential for cell elongation. Filaments of MreB rotate around the short axis of the cell, driven by peptidoglycan synthesis (van Teeffelen et al., 2011), and certain mutations in MreB cause irregular cell shape and/or altered, most often increased cell diameter (Shi et al., 2018; Kurita et al., 2019). By contrast, FtsZ is essential for cell division. It moves around the cell division plane by treadmilling to organize the divisome complex that synthesizes the new cell poles during cell division (Bisson-Filho et al., 2017; Yang et al., 2017).

In this *Perspectives* article we hypothesize that cytoskeleton dynamics and PG biosynthesis function to maintain cell dimensions in response to a yet elusive, primary and perhaps mechanical signal to determine cell width, a process that starts during physiological transitions at the new cell pole. Some compelling evidence indicates that such an early signal is related to the Bacterial Cell Division Cycle, so-called the BCD dogma (Helmstetter et al., 1968). A brief description of this dogma is provided below.

## The Bacterial Cell Division Cycle

Prokaryotic chain-synthesis rates of the three macromolecules involved in the flow of genetic information are constant, at least at 37°C, independent of the total mass growth rate. Transcribed mRNA is simultaneously co-translated with its gene sequence at modulus 3-matched rates, roughly 17 amino acids and 50 base pairs per second, respectively, by polysomes (Maaløe and Kjeldgaard, 1966). Another essential hyper-structure (Norris et al., 2007), the replisome replicates the 4.6 Mbp circular chromosome bidirectionally from *oriC* to *terC* as fast (O’Donnell et al., 2013) as 2,000 bp sec^-1^, over 10-fold faster than mammalian DNA replication fork, and with an amazingly low frequency of 10^-8^ mistakes. The times taken to complete (a) a round of replication *C* and (b) the subsequent cell division *D* are roughly constant, about 40 and 20 min, respectively, regardless of the total mass doubling time *τ_m_* (under 60 min at 37°C) (Helmstetter et al., 1968; Jiménez-Sánchez, 2018). Replication round is initiated when cell mass reaches a value *Mi* per *oriC* number (Donachie, 1968; Pritchard et al., 1969), at the mother cell (i.e., *cell cycles* overlap) when *τ_m_* < (*C+D*). At faster growth rates, when *τ_m_* < *C*, a new replication cycle inaugurates at *oriC* before the previous one ends at *terC* (i.e., *replication cycles* overlap), thus forming multi-forked replicating chromosomes, sometimes (when *τ_m_* < ∼ 20 min) (Taheri-Araghi et al., 2014) even at the grandmother’s cycle. (See Appendix for definitions of parameters and field-specific terms.)

## Bacterial Dimensions and the Cell Division Cycle

Faster growing cells are larger because they divide a constant time [(*C*+*D*) = ∼60 min] after initiating the chromosome replication (Helmstetter et al., 1968) at a constant mass *Mi* (Donachie, 1968; Pritchard et al., 1969; Amir, 2014). Replication is *linear* whereas mass [or volume, since density is constant (Kubitschek et al., 1984)] is synthesized *exponentially* at a rate inversely proportional to *τ_m_* (Koch, 1993). The dissociation between rates of growth and of replication was confirmed by extending *C* (slowing replication) by limiting the concentration of thymine [T] supplied in the growth medium of thymine-requiring mutants (Pritchard and Zaritsky, 1970). It was reassuring to find that this so-called thymine limitation results in larger average cell mass (Zaritsky and Pritchard, 1973) consistent with *M* = ln2 ×*Mi* × 2^(^*^C^*^+^*^D^*^)/^*^τ^*. This increased size of cells growing at an identical rate was anticipated *a priori* to be manifested by longer cells because they usually grow by elongation only; it was highly surprising to find that such thymine-limited cells are wider as well, just as faster growing cells are (Zaritsky and Pritchard, 1973). The common denominator to the two conditions at which cells are wider, shorter *τ_m_* at a constant *C* and longer *C* at a constant *τ_m_*, is the number of replication positions (Sueoka and Yoshikawa, 1965) *n* = *C*/*τ*. This parameter was used to define nucleoid complexity *NC* (Zaritsky et al., 2006) the culture-average amount of DNA in genome equivalents associated with a single *terC* (Woldringh et al., 1990) *G*/*terC*# = (*τ*/*C* × ln2) (2*^C^*^/^*^τ^*—1) = (2*^n^*—1)/(*n* × ln2); larger *NC* implies a larger nucleoid. The satisfactory correlation observed between cell width *W* and *NC* (Zaritsky, 2015; Campos et al., 2018) led to the idea (Zaritsky and Woldringh, 2015; Zaritsky et al., 2017) that cell length *L* is passively determined by the exponential rate of mass synthesis ( = volume growth) and active regulation of cell width by a putative signal that is transmitted from the nucleoid to the PG-synthetic machinery.

## Division Rate and Width: Changes During Nutritional Shift-Up

The classical experiment of nutritional shift-up to faster growth (Kjeldgaard et al., 1958) discovered temporary and orderly dissociations between the main synthetic activities, the most striking of which is the so-called “rate maintenance” of cell division that keeps at the pre-shift speed for about 65 min. This phenomenon was readily explained by the results that led to the BCD dogma (Helmstetter et al., 1968), and clarified the dependence of *M* and *G* on *τ_m_* under constant *Mi, C* and *D* (Donachie, 1968; Pritchard et al., 1969). Studying cell dimensions during a similar transition (Woldringh et al., 1980) likewise disclosed that the change in cell width occurs exclusively *during* the division process and *at* the division site: *L* continues to extend at the pre-shift rate until the first division, during which process (and only then) cell width *W* rises as well, locally at the divisome. This local change results in temporary pear-shaped, tapered cells (Zaritsky and Woldringh, 2015; Zaritsky et al., 2017). The new *W* equalizes along *L* and during the following growth and divisions thus recovering their straight cylindrical shape with new dimensions that fit the post-shift *τ_m_*; the equalizing process is slow likely because the adjustment in the widths of the net-like sacculus requires growth of the cell (Höltje, 1998).

Thus, two types of PG-synthetic activities must exist in separate hyper-structures (Norris et al., 2007): elongasome, operating along the cylindrical PG during *L*-extension, that keeps cell width constant, and divisome that operates perpendicularly during cell division and allows changes in *W* (Van der Ploeg et al., 2003).

## Temporal and Spatial Aspects of the Divisome and Elongasome Activities

Normal cell division is regulated by the nucloid, both temporally and spatially (Zaritsky and Woldringh, 2015; Zaritsky et al., 2017): it happens *after* chromosome replication is terminated and precisely *between* the two segregating nucleoids. Similarly, cell width *W* seems to be determined by the nucleoid in both arenas: it rises *during* the division process—and *at* the divisome only.

The mechanism that blocks cell division before two equal sets of its full genetic information are available by completing replication is under intense investigations (Männik and Bailey, 2015), but the one fixing *W* is neglected, likely due to the enormous variation among species, even strains within *E. coli* (Begg and Donachie, 1978). Regulation of cell width is likely complex due to the large number of downstream processes leading from the presumed primary signal(s) to the executing PG synthesizing machinery (Egan et al., 2017). Many mutants in the genes coding for the involved proteins would therefore change these pathways and hence, cell width or shape. For example, certain mutants with changes in MreB, which result in an altered helical pitch angle of the MreB cytoskeleton, have larger cell widths (Ouzounov et al., 2016). It was not reported if these cells have changed their growth rate or *NC*. It is also not known if cells with poorly functioning MreB or cells grown with sublethal concentrations of the MreB inhibitor A22 (Ouzounov et al., 2016) correlate their (abnormal) cell width with *NC* within a range of growth rates, as do cells with unaltered MreB. The widths of *B. subtilis* cells can be altered by increasing the level of certain PG synthases, which does not change the growth rate of the cells (Dion et al., 2019). Again, it is not known if cells disturbed in this way maintain the correlation between *NC* and cell width at different growth rates.

## Nature of the Primary Signal—Need for a New Paradigm

Various ideas have been entertained as signals to initiate the biochemical cascade of reactions leading to activation of the divisome, all in the realm of regulatory molecules (Egan and Vollmer, 2013; Du and Lutkenhaus, 2017). A physical element is preferred (Rabinovitch et al., 2003; Zaritsky and Woldringh, 2015), by analogy to the mode of thinking that brought about the “enzyme-cannot-make-enzyme (e-c-m-e) paradox” (Stent, 1968; Stent and Calendar, 1978): based on the knowledge in the 1930s, the omni-potent, highly variable proteins were seriously considered as the store of genetic information whereas the monotonous structure of DNA led the scientists to think of it as a mere reservoir of nucleotides. The theoretical “e-c-m-e paradox,” together with convincing discoveries of DNA structure and function resulted in The Central Dogma of Molecular Biology and understanding the flow of genetic information unidirectionally from nucleic acids to proteins (Stent, 1968). The structure of prokaryotic DNA as the bacterial nucleoid and convincing physiological studies (Helmstetter et al., 1968) exposed coupling between its replication / segregation with duplication of the other unique cellular macromolecule / structure, the PG sacculus, by cell division. The yet-to-be-disclosed mechanism that governs this coupling seems to need a new concept, one that is external to the never-ending search (e.g., Männik et al., 2018) for the primary signal by a “regulator-of-the-regulator paradox.” In other words: what is the nature of the division regulator that is at the top of the hierarchy? The template feature came from another discipline (information science) than chemistry (producing an enzyme); by analogy, triggering cell division may stem from physics—or another discipline that we are not aware of currently rather than the proteins involved in the division process itself. Can the divisome activation be triggered by the nucleoid’s complexity or replication status?

Two articles (Knox and Funk, 2014; Knox, 2018) introduced biophysical signaling “as having a central role in cancer through influences on cell proliferation, cell cycle progression, apoptosis, cell migration and orientation, as well as cell differentiation.” Moreover, “Many aspects of the cell cycle and systemic functioning are regulated by biophysical (bioelectric) signals. These include cell division and proliferation, embryonic development (e.g., left-right body asymmetry, axon outgrowth), epithelial wound healing, tissue regeneration and cancer cell migration.” It is proposed here that in bacteria too biophysical cues take major roles in signaling basic functions such as cell division and width determination at the divisome.

A hypothetical physical signal, transmitted from the nucleoid to the divisome that simultaneously activates cell division and determines cell width has been invoked as *primary* (Rabinovitch et al., 2003), involves the so-called “transertion” process (Woldringh, 2002): co-transcriptional translation of membrane protein genes coupled to insertion of these proteins to the membrane. The envelope is thus pulled toward the nucleoid and stressesed along cell length in a direction that changes during the last stage of the replication cycle. This change is presumed to be sensed by the cell to trigger the assembly of the divisome (Rabinovitch et al., 2003).

## Thymine- Limitation and -Step Transitions

Immediate reaction to [T]-step-down in thymine concentration slows replication (Pritchard and Zaritsky, 1970) (extends *C*) hence reduces division frequency due to postponed terminations of ongoing replication cycles. Continued exponential mass growth at identical rate of divisome-delayed cells results in larger *M* and temporarily longer cells (Zaritsky and Pritchard, 1973), just as nutritionally up-shifted cells overshoot their new steady-state *L* (Kjeldgaard et al., 1958). In both cases, the default mass growth is accommodated by the continued function of the elongasome, and *W* starts to rise later (Zaritsky et al., 2017), when the deferred divisomes are assembled following the delayed terminations. At faster growth of Thy^+^ strains, the longer time to complete the larger division septum is compensated by the faster rate of septum build-up, culminating by a manifested constant *D* period (Zaritsky et al., 1999). In contrast, compensation does not exist at identical growth rate in cells that are wider due to slow replication, and hence *D* is longer. A new steady-state is predicted to arise, with bigger cells due to the extended *D*. This scenario indeed happens under slower growth, in glycerol-supplemented minimal salts medium (*τ_m_* > ∼60 min).

This “simple” scenario, however, does not occur in glucose-supplemented, relatively fast-growing (*τ_m_* < ∼50 min) *thyA* mutants under thymine limitation (at low [T]) (Zaritsky and Pritchard, 1973). Under such circumstances, cells do not reach steady-state dimensions: the mean inter-division time *τ_d_* is longer than the constant *τ_m_*, hence mean culture cell size increases at a rate d*M*/dτ that is proportional to (*τ_d_*–*τ_m_*), which in turn depends inversely on the value of *C* that is [T]-dependent. Qualitatively, this phenomenon was explained by a sort of vicious circle (Zaritsky et al., 2017): extended *D* due to wider cells results in larger cells, that in turn further extends *D*, but this explanation completely ignores the question posed here: what causes the divisome in the first place to build a wider circumference when the number of replication positions *n* = *C*/*τ* is larger.

The slow rise in cell width *W* under these circumstances is, however, limited to a maximum, at which the increased cell size is accommodated by branching as well as extended *L* (see e.g., Figure 2 in Zaritsky et al., 2007). Putative link between *NC* and *W* may resolve this fundamental question; it predicts a maximum achievable width because of so-called eclipse effect, as described below.

## The Eclipse Concept

The unusual large and monstrous-shape of cells that slowly evolve during long periods of growth under this non-steady state conditions is reversible, and very fast, too: restoring replication rate using the thymine step-up regime temporarily but markedly enhance the frequency of divisions (Zaritsky et al., 2011); while *τ_m_* remains constant, ∼40 min at all [T] (above a certain strain- and medium -dependent threshold) (Zaritsky and Pritchard, 1971, 1973), divisions proceed almost synchronously at ∼20 min intervals for several cycles (at least 5 under the recorded circumstances). This is consistent with existence of another phenomenon: a minimal physical distance *l_min_* along the chromosome length *Λ* needed for a replisome to be away from *oriC* before a new replisome can initiate a subsequent replication round there (Zaritsky et al., 2007). In units of time, this so-called eclipse period *E* is proportional directly to the fraction *l_min_*/*Λ* and to *C*, the time taken to replicate the whole *Λ*, i.e., *E* = *C*(*l_min_*/*Λ*). This idea was originally conceived by observing results of totally different nature (Zaritsky, 1975b), substantiated and coined as eclipse a decade ago (Zaritsky et al., 2007), and experimentally confirmed recently by others (Khan et al., 2016).

To sum up, there are two seemingly contradictory observations: (a) rise, albeit slow, in cell width *W* during thymine limitation of fast growing *thyA* strains and (b) a limit to *W* that cause cells to elongate and branch when breached. These two are qualitatively consistent with existence of, respectively, (a) regulation of cell width by nucleoid complexity *NC* (symbolized as *NC*→*W*) that is affected at the divisome during the division process only, and (b) a maximum value for *NC* that is limited by the eclipse *E*. The latter is manifested under slow replication rate at low [T] ‘s, when *τ_m_* << *C*, (e.g., 2*τ_m_* < *C*), conditions that set a maximum number of simultaneously acting replisomes on a nucleoid resulting in *E* = (*τ_d_*–*τ_m_*) > 0. Thus, if indeed *NC*→*W, E* serves as a tool to manipulate (a) and explain (b), at least partially and qualitatively.

The *NC*→*W* hypothesis gains support from a recent article (Campos et al., 2018), suggesting “that the size of the nucleoid is an important element of the coordination mechanism between cell morphogenesis and the cell cycle.” The comprehensive data included may contain mutants which deviate from the model and so would point to proteins that are involved in the downstream processes of the mechanism involved.

## Can Gene Dosage Explain the *NC*→*W* Hypothesis?

*NC* is also reflected by gene dosage. Two quantities can be distinguished in a steady state cell population: *gene concentration* is the number of genes per cell mass (or volume) 2^-^*^nx^*/*Mi*, where *x* is the distance of a gene from *oriC*, and *relative gene dosage*, defined as the number of gene copies per total genome content 2*^n^*^(1-^*^x^*^)^/*NC* (Chandler and Pritchard, 1975). Both quantities depend exponentially on the gene location on the chromosome. Concentrations of all genes except *oriC* decrease as *NC* rises. The decrease depends on the position of the said gene on the genetic map; it drops as the gene is further downstream *oriC* and is largest for *terC*. The numbers of *oriC*-proximal genes are enriched compared to those of *terC*-proximal genes as *NC* rises. It was recently argued that relative gene dosage is the relevant parameter that determines the gene’s expression level (Lin and Amir, 2018). This argument stems from the assumption that the protein-synthesizing-system is rate-limiting for gene expression in *E. coli*. This assumption has, however, not been thoroughly tested yet. Thymine-limited cells could easily be used to test and advance these ideas further.

Variation of gene dosage across genome at different growth conditions can lead to changes in expression profiles of genes involved in cell wall synthesis, many of which are scattered, including *oriC*-proximal regions. Composition and cross-linking of septal cell wall can be expected to be affected by differential production levels of such cell wall synthesizing enzymes. Future proteomic or ribosome profiling studies (Li et al., 2014) may be used to test these hypotheses.

## Concluding Remarks

This manuscript deals with a fundamental question in bacteriology, relating cell dimensions and division to its nucleoid structure. The presumed signal(s) transmitted from the nucleoid (DNA) to its sacculus (PG) is crucial because both structures (macromolecules) are singular in a bacterium and essential for survival of the species. We suggest a function for the chromosome other than in replicating the genetic information and its flow from DNA to proteins.

The field of bacterial physiology, established in 1958, has since relied on genetics and chemistry. Involvement of physical forces at a higher (structural) level than sheer interactions between molecules, hardly dealt with in the literature, may open a new avenue to understand living matter. Participation of such forces in this signal needs pursuit. During the 20th century, physicists have been highly instrumental in advancing the quantitative biological sciences, e.g., the phage group led by Max Delbrück. Our call here is a far cry in the hope to devise methods that would measure the tiny intra-cellular forces, likely to take part in the functions discussed, cell division and shape determination.

Slowing replication rate by thymine limitation is a powerful tool to breach the minimal distance between successive replisomes with no detectable change in mass growth rate hence presumably cellular protein profile. In fast growing thymine-limited *thyA* mutants, further divisions and replication-initiations are delayed due to postponed replication-terminations and breaching the minimal distance—cumulatively so. The cascade of consequent reactions affect cell dimensions, overshooting length by default continuous elongasome activity initially, then width during the divisome activities until reaching a maximum that seems to be related to *NC*. The aberrant behavior of such cells can be exploited to decipher the mechanism(s) regulating the essential coupling between mass growth and the various biosynthetic activities of the nucleoid and the sacculus, and to discover the presumed primary signal relayed from DNA to the PG biosynthetic pathway in the divisome to simultaneously execute the division process and fix cell width. It is noteworthy that the postulated signal relayed from the nucleoid to the divisome may rather be related to the changes in gene concentration or relative gene dosage that reflect *NC*. Another alternative, testable theory (Gray et al., 2019), is that different nucleocytoplasmic ratios “can lead to different biophysical properties of the cytoplasm and hence to affect the mobility of large cytoplasmic objects.” This theory and our *NC*→PG hypothesis need further and new experimental approaches to define the underlying physical and molecular mechanisms. We reckon that time will tell whether any of these hypotheses is closer to the “truth” (i.e., reality). The detailed, complicated picture summarized here is consistent with the existing observations, though meager at this stage of knowledge.

## Author Contributions

All authors listed have made a substantial, direct and intellectual contribution to the work, and approved it for publication.

## Conflict of Interest Statement

The authors declare that the research was conducted in the absence of any commercial or financial relationships that could be construed as a potential conflict of interest.

## Appendix – Glossary: Parameters and Definitions of Field-Specific Terms Used (The Cell Cycle and Dimensions)

### Cell Growth and Cycle Parameters

*τ*, doubling time; *τ_m_*, of mass; *τ_d_*, between successive divisions *C*, replication time, taken to duplicate the entire chromosome, from origin *oriC* to terminus *terC* *D*, time between replication-termination and subsequent cell division *Λ*, chromosome length in genome equivalents units *E*, Eclipse - minimal possible distance *l_min_* along the chromosome length *Λ* needed for a replisome to be away from *oriC* before a succeeding replisome can initiate a next replication round there. In units of time, *E* is proportional directly to the fraction *l_min_*/*Λ* and to *C*, i.e., *E* = *C*(*l_min_*/*Λ*) *Mi*, initiation mass - cell mass per number of *oriC* at the time of replication-initiation *n*, number of replisome positions, equal to *C*/*τ* *NC*, nucleoid complexity - amount of DNA in genome equivalents associated with a single *terC*, equals to (2*^n^*—1)/(*n* × ln2) *x*, distance of a gene from *oriC* in units of *Λ*

### Cell Dimensions and Composition

*M*, average cell mass in a steady-state culture growing in batch, equal to ln2 × *Mi* × 2^(^*^C^*^+^*^D^*^)/^*^τ^* *L*, cell length *V*, cell volume *W*, cell width *G*, amount of DNA per cell in genome equivalents *PG*, peptidoglycan [T], concentration of thymine supplied to growth media of *thyA* mutants

### Hyperstructures and Processes

*Divisome*: A contractile ring of polypeptides involved in bacterial cell division *Replisome*: A matrix of enzymes that is the site of DNA replication *Transertion*: Co-transcriptional translation of membrane protein genes coupled to insertion of these proteins to the membrane
